# Case Report: A case of uterine embryonal rhabdomyosarcoma in adult female—navigating the complexities of the diagnostic journey

**DOI:** 10.3389/fonc.2025.1535933

**Published:** 2025-07-09

**Authors:** Sijing Li, Dongni Zhou, Qiao Zu, Ying Jia

**Affiliations:** 1 Department of Obstetrics and Gynecology, The First Affiliated Hospital of Chongqing Medical University, Chongqing, China; 2 Department of Obstetrics and Gynecology, Women and Children’s Hospital of Chongqing Medical University, Chongqing, China; 3 Department of Obstetrics and Gynecology, Chongqing Health Center for Women and Children, Chongqing, China

**Keywords:** rhabdomyosarcoma, adult embryonal rhabdomyosarcoma, diagnosis, cervix, treatment

## Abstract

Rhabdomyosarcoma (RMS) is a soft tissue sarcoma originating from primitive mesenchymal cells that exhibit varying degrees of skeletal muscle differentiation. Although RMS predominantly affects children and adolescents—representing one of the most common pediatric solid malignancies—it is exceptionally rare in adults, constituting less than 1% of adult cancers. Due to its embryonic mesenchymal origin, RMS can develop in virtually any organ. In adults, the most common sites are the trunk (27%) and limbs (26%), with the reproductive tract accounting for 17%. Current understanding of adult embryonal RMS primarily relies on case reports, as it is often misdiagnosed as other benign or malignant solid tumors. Here, we report a case of botryoid RMS of the uterine cervix in an adult woman. The diagnostic process was prolonged; after five visits and four biopsies, the diagnosis of rhabdomyosarcoma was finally confirmed. Based on pathological findings and imaging examinations, the clinical stage was determined to be T1N0M0 and Intergroup Rhabdomyosarcoma Study (IRS) IA. The patient was initially scheduled for surgery following neoadjuvant chemotherapy. However, after one cycle of chemotherapy, she experienced massive vaginal bleeding and prolapse of the cervical polypoid mass. Due to significant psychological distress, the patient declined further chemotherapy and insisted on proceeding with surgery. Subsequently, she underwent a robot-assisted laparoscopic radical hysterectomy, bilateral salpingo-oophorectomy, and pelvic lymph node dissection. A total of six cycles of the VA chemotherapy regimen were administered both pre- and postoperatively. Unfortunately, the 8-month postoperative follow-up results were unfavorable. Less than a year after surgery, contrast-enhanced pelvic MRI revealed enlarged pelvic lymph nodes, suggesting a possible recurrence. The purpose of this study was to report a case of embryonal rhabdomyosarcoma (ERMS) of the uterine cervix in an adult woman and to highlight the diagnostic and therapeutic challenges associated with this condition.

## Background

Rhabdomyosarcoma (RMS) is a soft tissue sarcoma that originates from primitive mesenchymal cells showing varying degrees of differentiation into skeletal muscle (1). There are four major histologic subtypes of rhabdomyosarcoma: embryonal rhabdomyosarcoma (ERMS), alveolar rhabdomyosarcoma (ARMS), pleomorphic rhabdomyosarcoma (PRMS), and spindle cell/sclerosing rhabdomyosarcoma (SRMS) (2). Botryoid rhabdomyosarcoma is a special subtype of embryonal rhabdomyosarcoma. RMS predominantly affects children and adolescents, representing one of the most common pediatric malignant solid tumors and accounting for 5% to 10% of all childhood tumors (3). In adults, the incidence of RMS is approximately 0.1 to 0.3 cases per one million individuals annually (4). Given its embryonic mesenchymal origin, RMS can develop in virtually any organ. The primary sites of adult rhabdomyosarcoma are the trunk (27%) and limbs (26%), followed by the reproductive tract (17%). Most RMS cases in the female reproductive tract are ERMS, with the botryoid subtype being particularly notable. ERMS accounts 0.2% of malignant uterine tumors in adult women (4). Nearly all current information on adult ERMS is derived from case reports. Due to the extreme rarity and variable clinical manifestations of cervical ERMS, patients are often overlooked or misdiagnosed during the initial evaluation. In this report, we present a case of ERMS of the uterine cervix in an adult woman who experienced a complex and prolonged diagnostic process. The purpose of this study is to report this rare case and to highlight the diagnostic and therapeutic challenges associated with this condition.

## Case report

The patient was a 41-year-old woman with regular menstrual cycles and a history of one live birth from seven pregnancies. She presented to our hospital with a 9-month history of abnormal vaginal bleeding. In June 2022, she experienced postcoital bleeding and sought treatment at a primary hospital, where colposcopy and hysteroscopy revealed a cervical polypoid mass. The mass was resected, and the excised tissue was sent for pathological examination, which initially suggested cervical polyps. The patient continued to experience recurrent vaginal bleeding after surgery and repeatedly visited the primary hospital for treatment. During this period, the cervical mass progressively increased in size. In September 2022, a repeat biopsy of the cervical mass was performed, but the pathological findings once again indicated cervical polyps.

**Figure 1:**
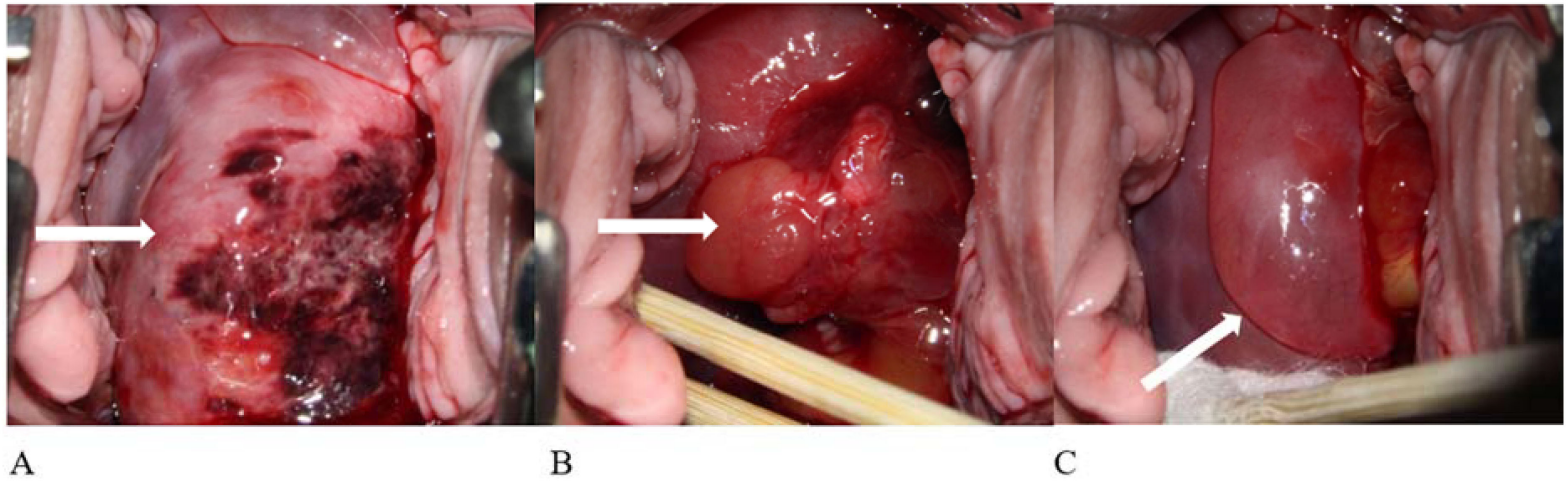
Colposcopy images of the patient taken in February 2023. A large amount of blister-like, grape-shaped tissue was present in the vagina, accompanied by numerous blood clots. As these obstructed cervical visualization, colposcopy was performed after removing the detached blister-like tissue and blood clots. **(A)** Cervical polypoid vegetation with necrotic foci. **(B)** Obvious polypoid tissue, blood clots, and blister-like formations. **(C)** Large blister-like tissue.

**Figure 2:**
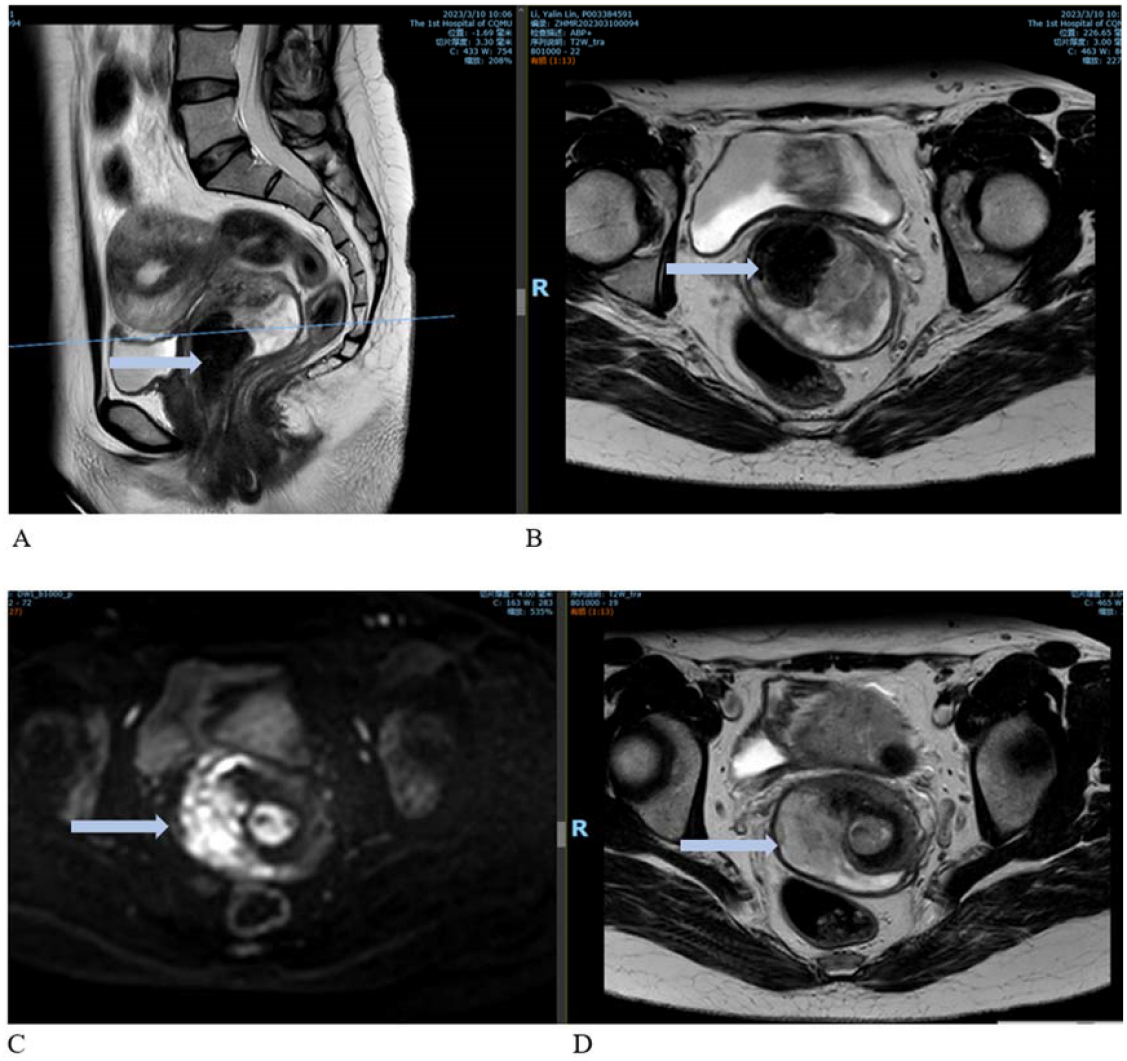
Contrast-enhanced MRI images. **(A)** Sagittal view showing an enlarged cervix with an irregularly shaped mass extending to the internal cervical orifice and into the vagina. **(B)** Cross-sectional view of the lesion. **(C, D)** Enhanced images showing marked enhancement at the cervical lesion site.

**Figure 3:**
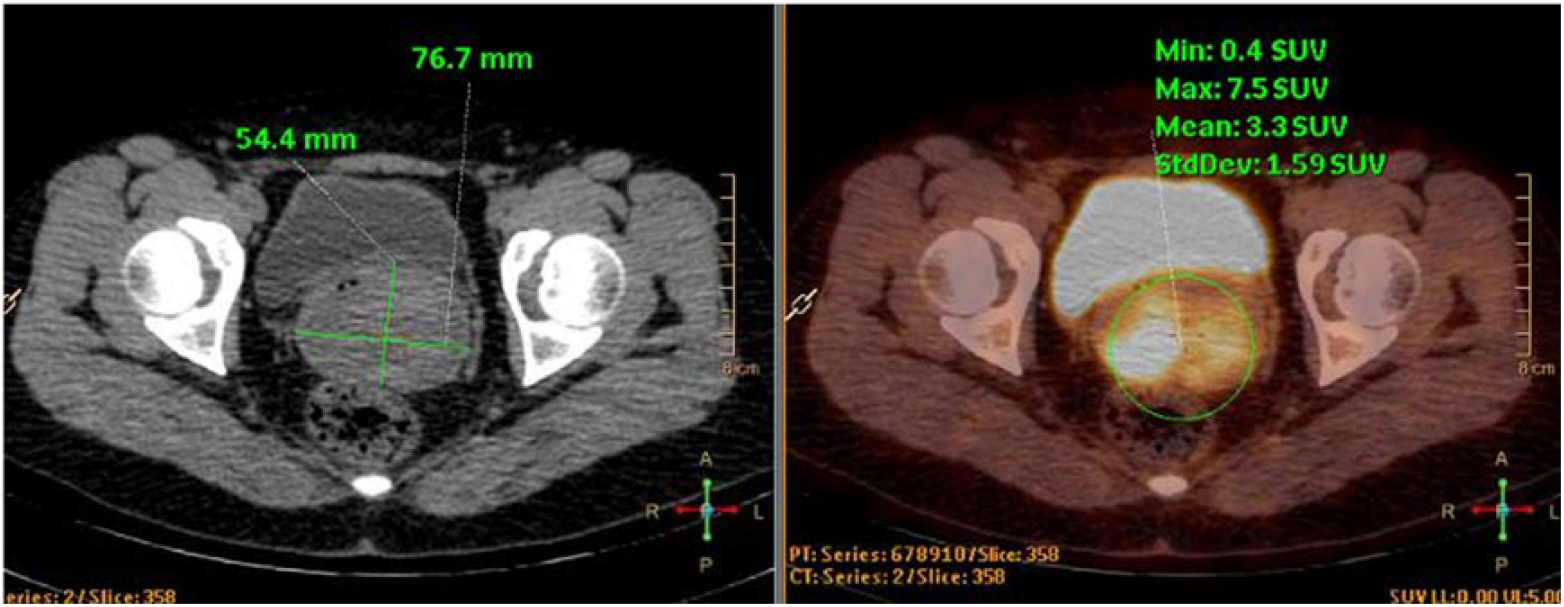
PET-CT images. The cervical lesion showed increased metabolic activity, consistent with features of a malignant tumor.

The patient was initially evaluated at our center in February 2023. Gynecological examination revealed numerous grape-shaped, blister-like tissues protruding from the external os of the cervix, with obvious contact bleeding. Colposcopy showed prominent polypoid vegetation originating from the middle and upper portions of the vaginal wall. The mass had a soft texture and was accompanied by necrotic tissue and blood clots. Numerous blister-like tissues were observed surrounding the mass (**Figure 1**). Biopsy of the cervical lesion indicated that the cervical vegetations and blister-like tissues contained clusters of atypical cells, suggestive of a neoplastic lesion. Immunohistochemistry showed the following: P16 (−), p40 (−), and Ki-67 (+) about 20%. Special staining showed positivity (+) for reticular fibers. There were no significant abnormalities in tumor markers (e.g., CA125, HE4, CA199, SCC). Enhanced MRI revealed an enlarged cervix with an irregularly shaped mass extending to the internal cervical os, without endometrial involvement. The cervical stromal ring was incomplete, and the lesion extended into the vagina. The parametrial tissue remained unaffected, and the vaginal morphology and signal appeared normal. Both fallopian tubes and ovaries were unremarkable bilaterally, and no enlarged pelvic or abdominal lymph nodes were observed (**Figure 2**). PET-CT demonstrated cervical enlargement with lesion extension into the adjacent vagina and showed increased metabolic activity, consistent with malignant tumor characteristics (**Figure 3**).

One month later (March 2023), the patient returned to our center for follow-up. On physical examination, a large amount of blister-like tissue was observed prolapsing from the vagina. The prolapsed tissue was excised and sent for pathological examination, which confirmed rhabdomyosarcoma with anaplastic features, consistent with graphiform rhabdomyosarcoma. Immunohistochemistry showed the following: CK (−), CAM5.2 (−), CKL (−), CKH (−), EMA (−), SMA (−), desmin (+), MyoD1 (+), myogenin (+), myoglobin (−), Vimentin (VIM) (+), PAX5 (−), CD99 (−), NKX2.2 (−), WT1 (−), LCA (−), SS18 (−), Syn (−), CgA (−), and Ki-67 90% (+). After five visits and four biopsies, a diagnosis of rhabdomyosarcoma was finally established (the four biopsies are shown in **Table 1**).

**Table 1:** Summary of the patient’s four preoperative pathological biopsies.

Date of biopsy	Pathological biopsy results	The pathological results reviewed in our center
June 2022	Cervical polyp	Cervical polyp
September 2022	Cervical polyps. Local stromal cell proliferation was active. Immunohistochemistry: SMA: −, desmin: −, CD34: vascular +, myogenin: −, MyoD1: −, P53: −, Ki-67: + about 5%, S-100: −, P16 −, CK: +. A superficial angiomyxoma was considered.	Cervical polyp with glandular squamous metaplasia and focal low-grade intraepithelial lesion (CIN1). Immunohistochemically, the tumor cells were positive for P16, ER, PR, vimentin, CEA, desmin, SMA, and cyclinD1, and showed approximately 20% Ki-67 positivity.
February 2023	Abnormal cells were seen in the examined tissues, which were considered neoplastic lesions. Immunohistochemistry: P16 (−), p40 (−), and Ki-67 (+) at about 20%. Special staining: reticular fibers (+).	
March 2023	Embryonal rhabdomyosarcoma. Immunohistochemistry: CK (−), CAM 5.2 (−), CKL (−), CKH (−), EMA (−), SMA (−), desmin (+), MyoD1 (+), myogenin (+), myoglobin (−), VIM (+), PAX5 (−), CD99 (−), NKX2.2 (−), WT1 (−), LCA (−), SS18 (−), Syn (−), CgA (−), Ki-67 90% (+).	

**Figure 4:**
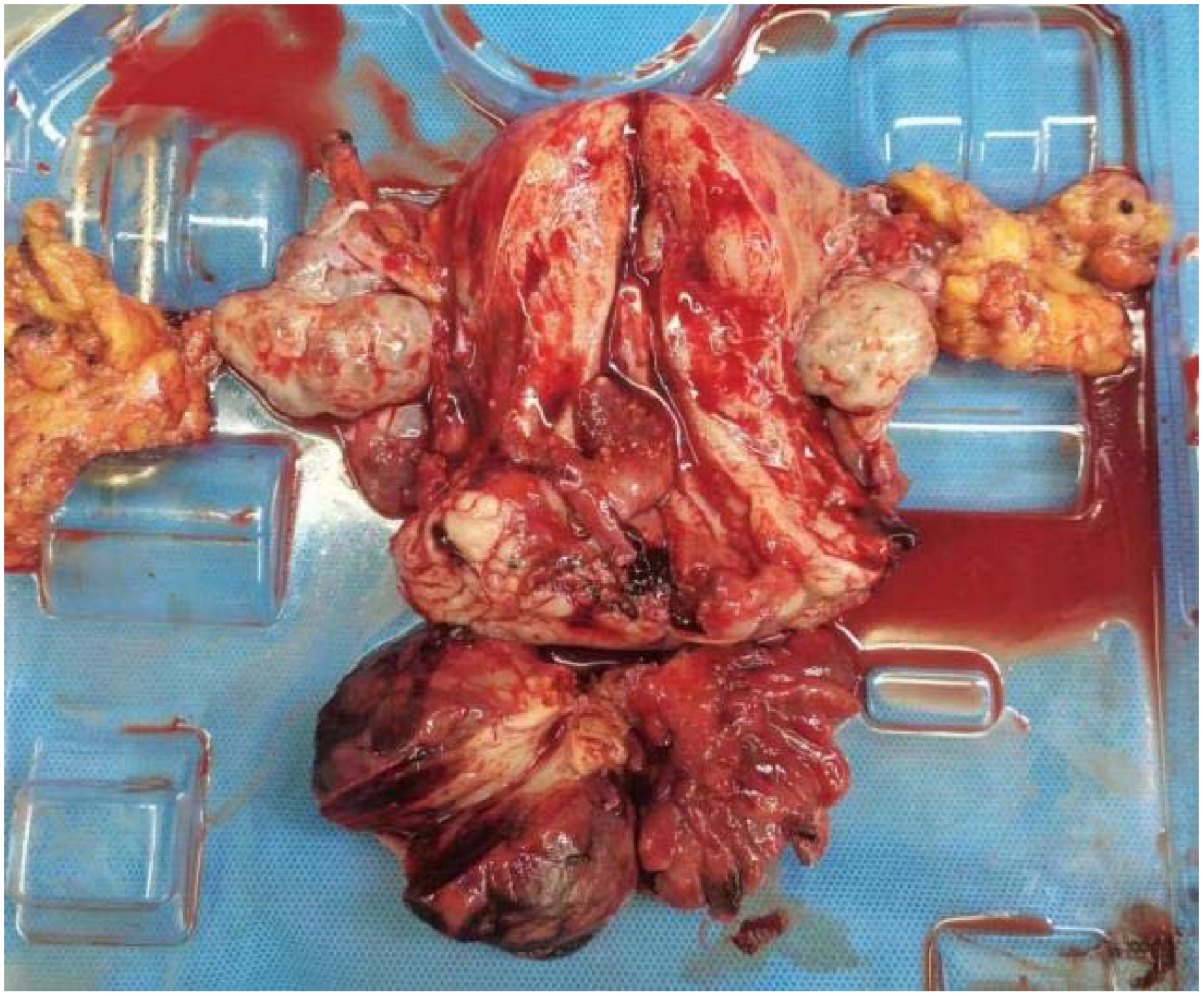
Postoperative specimen. The cervical vegetations appeared string-like, with roots located on the right posterior wall of the lower segment of the cervical canal. The vaginal wall was edematous and thickened, with no vegetation.

**Figure 5:**
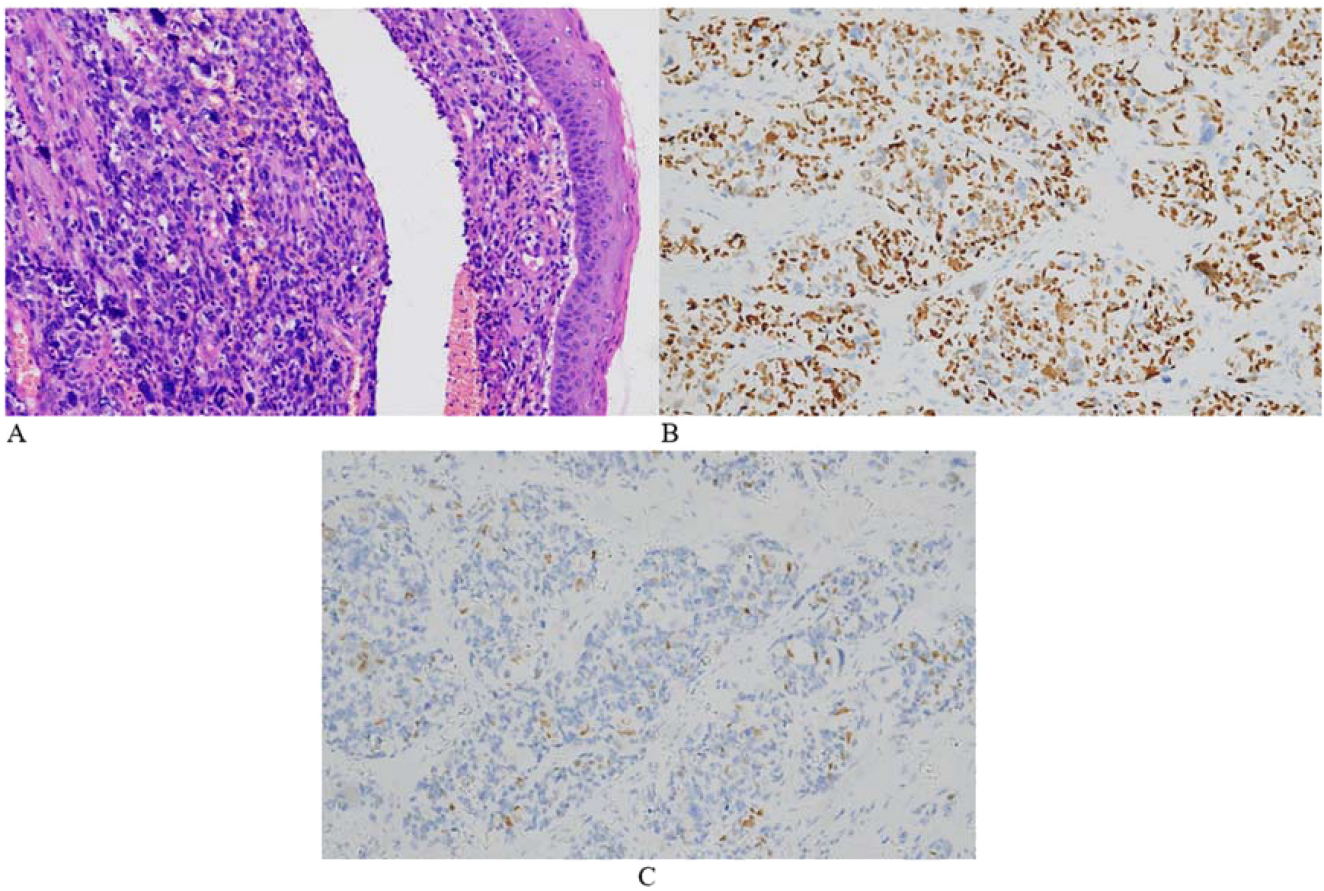
Representative microscopic images of the cervical lesion. H&E staining revealed obvious tumor cell atypia in some areas, uneven distribution of tumor cells, and large nuclei. The tumor exhibited giant cells with anaplastic changes and frequent mitotic figures (**A**, HE 200×). Immunohistochemical staining was positive for myoD1 **(B)** and myogenin **(C)**.

Based on pathological findings and imaging examinations, the patient was ultimately diagnosed with uterine embryonal rhabdomyosarcoma. The clinical stage was determined as T1N0M0 (tumor size > 5 cm) and IRS IA. Elective surgery was scheduled following 6 weeks of chemotherapy. The chemotherapy regimen included vincristine and actinomycin (VA). After the first cycle, the patient experienced heavy vaginal bleeding of unknown cause, accompanied by prolapse of vaginal tissue. Physical examination revealed a cervical polypoid mass measuring 6 × 5 × 5 cm that had fallen out of the vagina, with obvious contact bleeding. The patient was extremely anxious and completed only one cycle of chemotherapy before opting for surgery ahead of schedule. In April 2023, she underwent a robot-assisted laparoscopic radical hysterectomy, bilateral salpingo-oophorectomy, and pelvic lymph node dissection. The procedure was successful. Examination of the postoperative specimen revealed a uterine cavity depth of 9 cm with a flat endometrium. The cervical vegetations appeared string-like, with their roots located on the right posterior wall of the lower segment of the cervical canal. The vaginal wall was edematous and thickened but showed no vegetations (**Figure 4**). Postoperative pathological biopsy confirmed that the cervical neoplasm was embryonal rhabdomyosarcoma. Locally degenerated rhabdomyosarcoma tissue was identified in the outer half of the cervical wall. Immunohistochemical analysis revealed focal positivity for desmin and positive staining for MyoD1 and myogenin, while CK, VIM, and S100 were negative. The Ki-67 proliferation index was approximately 70% (**Figure 5**). No metastatic lesions were found in the bilateral fallopian tubes, ovaries, or pelvic lymph nodes. The uterine and vaginal walls were free of tumor involvement. Postoperatively, the patient received five cycles of intravenous chemotherapy using the VA regimen.

**Figure 6:**
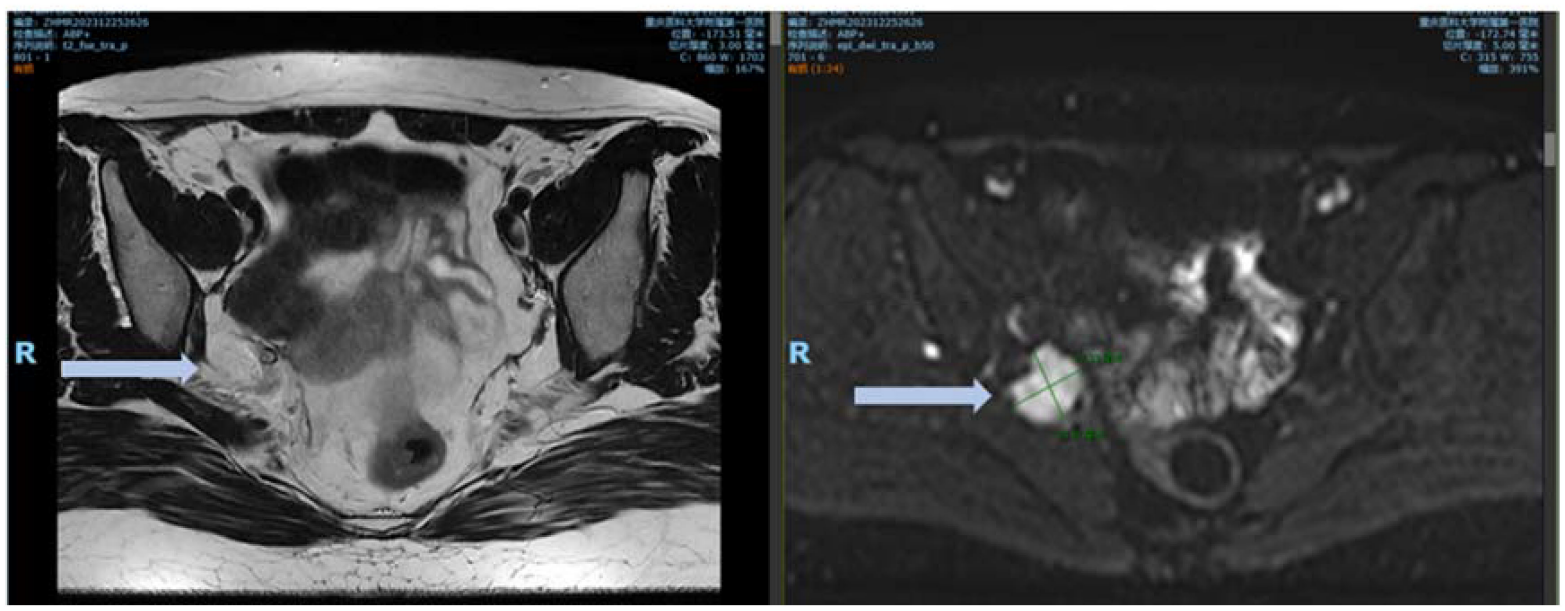
MRI images 8 months after surgery. DWI demonstrated an obvious high signal, with marked enhancement observed on the contrast-enhanced images.

At the 8-month postoperative follow-up at our center, MRI detected a 2.7-cm soft tissue nodule adjacent to the internal iliac vessels with indistinct boundaries. Pelvic contrast-enhanced MRI findings suggested a suspected recurrence (**Figure 6**).

## Discussion

Botryoid rhabdomyosarcoma is a distinct subtype of embryonal rhabdomyosarcoma. It often occurs in the genitourinary and respiratory tracts but is rare in the cervix. Tumors in the cervical region tend to be highly malignant. Adult cervical ERMS is a rare disease, with most available data limited to case reports. This case report highlights a challenging diagnostic journey, involving two misdiagnoses and transitions between two medical institutions over a prolonged 9-month period. This case aims to raise clinicians’ awareness of cervical ERMS, especially when evaluating patients presenting with abnormal vaginal bleeding, to ensure timely recognition and diagnosis of this rare condition.

The clinical symptoms of ERMS are nonspecific. In reported cases of cervical ERMS, most patients present with vaginal bleeding and a protruding polypoid mass. Typically, a friable polypoid mass or one covered by smooth mucosa is observed on the cervix. There are no effective auxiliary diagnostic methods for cervical ERMS, and biopsies often fail to detect the tumor. Due to its polypoid appearance, the tumor frequently displays multiple lobules. Microscopically, typical ERMS is characterized by normal mucosal epithelium covering the tumor surface, beneath which lies a dense “cambium layer” of tumor cells and rhabdomyoblasts at various stages of differentiation. Areas of hypercellularity around blood vessels alternate with hypocellular regions rich in myxoid matrix. Tumor cells are unevenly distributed, featuring large nuclei and variably eosinophilic cytoplasm. In some regions, tumor cells are loosely arranged within a myxoid stroma, and mitotic figures are frequently observed. Focal hyaline cartilage is present in 45% of cases (5), although it was not observed in this case. Tissue sections often yield negative results under microscopic examination due to insufficient pathological sampling, low density of sarcoma cells at the sampling site, or incomplete eosin staining, which can lead to misdiagnosis as cervical polyps. Ferguson and Lee et al. reported that up to 25% of women initially received an incorrect diagnosis, with the correct diagnosis established only upon recurrence (6).

In this patient, the initial two pathological examinations indicated “cervical polyps”. Upon review of these missed diagnoses, it was noted that immunohistochemistry was not performed during the first examination. Although immunohistochemistry was conducted during the second examination, desmin and myogenin staining were negative, and tumor proliferative activity was not significantly elevated. This discrepancy may be attributed to sampling errors, possibly due to hemorrhage in the lesion area preventing collection of cell-rich tumor tissue.

The application of immunohistochemistry plays an important role in the diagnosis of ERMS. In immunohistochemical staining, rhabdomyosarcoma (RMS) exhibits positive expression of vimentin, desmin, myogenin, and MyoD1. Vimentin is primarily used to differentiate carcinoma from sarcoma, demonstrating high sensitivity but low specificity. In contrast, MyoD1 and myogenin demonstrate both high specificity and sensitivity for diagnosing RMS, with positive staining localized in the nucleus, making them reliable diagnostic markers for rhabdomyosarcoma (7). Li et al. analyzed the clinicopathological and immunohistochemical features of ERMS in 25 adult women over 20 years old. Desmin and myogenin were expressed in 95.6% (22/23) of the tumors, and all tumors showed significantly increased proliferative activity (8).

Adult ERMS often invades the bladder, rectum, or other pelvic organs. However, despite a prolonged disease course of 9 months in this patient, the lesion remained confined to the cervix, without involvement of the parametrial tissue or adjacent organs.

RMS is sensitive to both chemotherapy and radiotherapy; however, single-modality treatment is often insufficient. Therefore, a combination of surgery, radiotherapy, and chemotherapy, along with long-term follow-up, is essential. Effective RMS treatment depends on accurate disease assessment. The Intergroup Rhabdomyosarcoma Study Group (IRSG) grouping criteria, widely used both domestically and internationally, classify RMS into risk groups based on tumor location, size, resection status, presence of metastasis, and pathological subtype (**Table 2**) (9). In the IRSG-V study, RMS risk is stratified into low-, intermediate-, and high-risk categories based on tumor pathology, grouping, and staging, which guides tailored treatment (**Table 3**).

**Table 2:** The surgical-pathologic grouping system used in IRSG trials.

Group No.	Criteria
Group I	Localized disease, completely resected Confined to the organ or muscle of origin Infiltration outside organ or muscle of origin; regional lymph nodes not involved
Group II	Compromised or regional resection of three types, including: Grossly resected tumors with microscopic residual; Regional disease, completely resected, in which lymph nodes may be involved and/or extension of tumor into an adjacent organ may be present; Regional disease with involved lymph nodes, macroscopically resected but with evidence of microscopic residual.
Group III	Incomplete resection or biopsy with macroscopic residual disease.
Group IV	Distant metastases present at onset.

IRSG, Intergroup Rhabdomyosarcoma Study Group.

**Table 3:** RMS risk stratification.

Risk stratification	Criteria
Low-risk	Groups I–II ERMS, group III ERMS in favorable prognostic sites
Intermediate-risk	Groups I–III ARMS at any site, group III ERMS at sites with poor prognosis
High-risk	Tumors with distant metastases

In this case, the patient achieved complete local resection with negative surgical margins, no regional lymph node involvement, and a diagnosis of ERMS, indicating a low-risk classification. Current treatment strategies for cervical ERMS are largely based on protocols for RMS at other sites and, according to the IRSG trials, primarily target pediatric patients. However, the poorer prognosis of ERMS in adults compared to children and young women requires a more aggressive treatment approach (10). A combination regimen of vincristine, actinomycin, and cyclophosphamide (VAC) is the standard chemotherapy for pediatric patients with nonmetastatic rhabdomyosarcoma (classified as intermediate or high risk). The Intergroup Rhabdomyosarcoma Study Group study reported that 5-year disease-free survival rates were comparable between patients with newly diagnosed, low-risk rhabdomyosarcoma treated with VA versus the full VAC regimen (89% vs. 85%) (11). This finding suggests that vincristine and actinomycin alone may be appropriate for patients with newly diagnosed, low-risk rhabdomyosarcoma. Therefore, the patient was treated with a VA chemotherapy regimen. Due to the patient’s lack of fertility requirements and high anxiety, an extensive total hysterectomy, bilateral oophorectomy, and pelvic lymph node dissection were performed. Six cycles of the VA chemotherapy regimen were administered both pre- and postoperatively. Unfortunately, the follow-up at 8 months postsurgery was not favorable. Less than 1 year after surgery, contrast-enhanced pelvic MRI revealed enlarged pelvic lymph nodes, suggesting possible recurrence.

RMS is also highly sensitive to radiotherapy. Concurrent chemoradiotherapy following chemotherapy is the standard treatment for unresectable tumors. Radiotherapy is considered for patients with low-risk ERMS who have residual disease after surgery or unfavorable primary sites where complete surgical resection is not feasible (12). The addition of radiotherapy to VAC chemotherapy improves local control rates, particularly in patients with microscopic or gross residual disease after surgery. In this case, postoperative pathological examination revealed no residual tumor, negative surgical margins, and no lymph node involvement; therefore, adjuvant radiotherapy was not administered.

Literature reports indicate that the recurrence rate of cervical ERMS in adults can reach 20%–40%, potentially due to occult micrometastases or tumor biological characteristics (e.g., fusion gene negativity and MYOD1 mutations). Most recurrences occur within 2 years postoperatively, although late recurrences (> 5 years) have also been reported (13). Among these cases, patients with tumors larger than 5 cm are at increased risk of adverse outcomes following recurrence (14). In the present case, the primary lesion exceeded 5 cm in diameter, thereby heightening the risk of recurrence. Although the surgical margins were negative, retrospective analysis suggests that adjuvant low-dose radiotherapy might have improved the patient’s prognosis.

In conclusion, cervical ERMS is a rare adult soft tissue malignancy in adults, characterized by significant clinical and biological heterogeneity, with nonspecific clinical manifestations. In this case, the patient underwent multiple evaluations before a definitive diagnosis was established. This challenging diagnostic process highlights the clinical similarity between cervical RMS and benign conditions, such as cervical polyps. Clinicians should increase their awareness of rare diseases such as cervical RMS, particularly when evaluating patients with “cervical vegetations” or “abnormal vaginal bleeding”, to ensure timely consideration of malignant conditions. In addition, clinical biopsies may yield insufficient samples or negative immunohistochemical results; therefore, it is crucial to submit adequate tissue specimens for pathological examination. At present, the treatment of this disease emphasizes individualized approaches. The main treatment options are surgery and chemotherapy. However, due to the rarity of these lesions, clinical experience is limited, and no expert consensus or guidelines are currently available. Further research is needed to establish optimal treatment strategies. We hope that this case report contributes to improving the diagnosis and management of this disease.

## Data Availability

The datasets presented in this study can be found in online repositories. The names of the repository/repositories and accession number(s) can be found in the article/supplementary material.
