# Correction: Case Report: A case of uterine embryonal rhabdomyosarcoma in adult female—navigating the complexities of the diagnostic journey

**DOI:** 10.3389/fonc.2025.1667442

**Published:** 2025-08-08

**Authors:** Sijing Li, Dongni Zhou, Qiao Zu, Ying Jia

**Affiliations:** ^1^ Department of Obstetrics and Gynecology, Women and Children’s Hospital of Chongqing Medical University, Chongqing, China; ^2^ Department of Obstetrics and Gynecology, Chongqing Health Center for Women and Children, Chongqing, China; ^3^ Department of Obstetrics and Gynecology, The First Affiliated Hospital of Chongqing Medical University, Chongqing, China

**Keywords:** rhabdomyosarcoma, adult embryonal rhabdomyosarcoma, diagnosis, cervix, treatment

Author Ying Jia was erroneously assigned to affiliation 2 “Department of Obstetrics and Gynecology, Chongqing Health Center for Women and Children, Chongqing, China”. This affiliation has now been removed for author Ying Jia and the correct affiliation 3 has now been added “Department of Obstetrics and Gynecology, The First Affiliated Hospital of Chongqing Medical University, Chongqing, China”.

The original version of this article has been updated.

