# Correction: Case Report: A case of uterine embryonal rhabdomyosarcoma in adult female—navigating the complexities of the diagnostic journey

**DOI:** 10.3389/fonc.2026.1788762

**Published:** 2026-01-29

**Authors:** Sijing Li, Dongni Zhou, Qiao Zu, Ying Jia

**Affiliations:** 1 Department of Obstetrics and Gynecology, The First Affiliated Hospital of Chongqing Medical University, Chongqing, China; 2 Department of Obstetrics and Gynecology, Women and Children’s Hospital of Chongqing Medical University, Chongqing, China; 3 Department of Obstetrics and Gynecology, Chongqing Health Center for Women and Children, Chongqing, China

**Keywords:** rhabdomyosarcoma, adult embryonal rhabdomyosarcoma, diagnosis, cervix, treatment

Affiliation “Department of Obstetrics and Gynecology, The First Affiliated Hospital of Chongqing Medical University, Chongqing, China” was omitted for author Sijing Li. This affiliation has now been added for author Sijing Li.

The original version of this article has been updated.

